# Mechanical and Thermal Behaviours of Weft-Knitted Spacer Fabric Structure with Inlays for Insole Applications

**DOI:** 10.3390/polym14030619

**Published:** 2022-02-05

**Authors:** Nga-Wun Li, Kit-Lun Yick, Annie Yu, Sen Ning

**Affiliations:** 1 Laboratory for Artificial Intelligence in Design, Hong Kong, China; dorisli@aidlab.hk; 2 Institute of Textiles and Clothing, The Hong Kong Polytechnic University, Hong Kong, China; sen-karolyn.ning@connect.polyu.hk; 3 Department of Advanced Fibro Science, Kyoto Institute of Technology, Kyoto 606-8585, Japan; annieyu@kit.ac.jp

**Keywords:** compression, thermal comfort, weft-knitted spacer fabric, inlay knitting, cushioning insole, silicone inlay

## Abstract

Insoles provide resistance to ground reaction forces and comfort during walking. In this study, a novel weft-knitted spacer fabric structure with inlays for insoles is proposed which not only absorbs shock and resists pressure, but also allows heat dissipation for enhanced thermal comfort. The results show that the inlay density and spacer yarn increase compression resistance and reduce impact forces. The increased spacer yarn density provides better air permeability but reduces thermal resistance, while a lower inlay density with a random orientation reduces the evaporative resistance. The proposed structure has significantly positive implications for insole applications.

## 1. Introduction

Insoles act as an important cushioning feature at the interface between the foot and footwear to manipulate the distribution of plantar pressure for foot protection and wear comfort. However, inappropriate insole designs and materials can lead to excessive moisture, heat and localized pressure points on the foot, which could then result in ulceration, arthritis or tendon dysfunctions. The growth of microorganisms in the enclosed environment of the inner shoe can also cause athlete’s foot or undesirable odours [1]. The literature on footwear research has mainly focused on the structural design and fabrication materials of insoles to relieve plantar pressure, and thus reduce the risk of ulceration [2,3], falls [4,5], and growth of microbes [1] as well as ensure the sustainability of the insole [6]. Recently, spacer fabrics have been used as an alternative cushioning material because this material offers a wide range of good properties, and has good compression behaviour, high density and adequate thickness for different applications, including insoles [7], helmet liners [8], wound dressings [9], sports bras [10], etc. The structure of spacer fabric is like a sandwich, with two separate layers of fabric on the outside and a connective layer between them [11]. In weft knitted spacer fabric, the connective layer is developed by using spacer filaments to form tuck stitches by using the front and back needle beds to create support for the two surface layers of fabric [12]. This sandwich structure provides spacer fabric with excellent air and moisture permeabilities, cushioning performance and pressure distribution [8,10,13,14,15].

Previous studies have found that the compression properties are significantly affected by the spacer yarns in the connective layer. The spacer fabric is stiffer in compression when the spacer filaments stand perpendicular to the layer of surface fabric [14]. On the other hand, spacer fabric has better compression resistance and energy absorption when there is a smaller span distance between the spacer yarns, coarser monofilaments, and higher spacer yarn density [15]. Hamedi et al. developed weft knitted spacer fabric to construct insole materials for diabetic patients by using a shape memory alloy, nickel-titanium, as the spacer fabric monofilaments [16]. However, spacer fabrics deform easily under high compression stress due to body weight and lose their cushioning properties after prolonged use. Also, the use of metal wire in knitting requires special equipment and is time consuming. Yu et al. proposed the use of inlays to reinforce the spacer fabric structure and found that the Young’s modulus of the inlaid silicone tubes is correlated with fabric compression [17]. Inlay knitting enables materials such as foam rods that usually cannot be knitted as part of the loops of the ground structure to be securely incorporated into the knitted fabric [18]. Its application can then be more widely used in the structural design of compression garments, buoyant swimwear, and sporting goods [19,20].

As for footwear and insole applications, increases in foot temperature incurred by the insoles during daily activities may result in a high level of foot discomfort from heat and perspiration. By taking the prolonged use and hygiene of insoles into consideration, the temperature and humidity transfer performance of insole fabrication material is regarded as the most important considerations for footwear comfort [21]. Transmission of moisture facilitates liquid to be evaporated through fabric to the environment which results in a cooling sensation for the wearer [22,23] and prevents a damp feeling which leads to discomfort. Nevertheless, few studies have focused on the thermal comfort of spacer fabric with inlays. While a preliminary study has shown that inlaid spacer fabric has superior air permeability [24], the impacts of the inlay density and orientation in the spacer fabric structure on the mechanical and thermal comfort properties have been largely absent from discussion. A more in-depth understanding of the effects of the inlay structure of spacer fabric and knitting parameters on mechanical and thermal behaviours is therefore paramount. The fabric structural parameters that affect the compression behaviour, impact force reduction, and thermal and evaporative resistance are systematically analysed. The findings of this study can greatly contribute to foot protection and orthotic treatment by providing insights into suitable insole materials.

## 2. Materials and Methods

### 2.1. Preparation of Inlaid Foam

**Figure 1:**
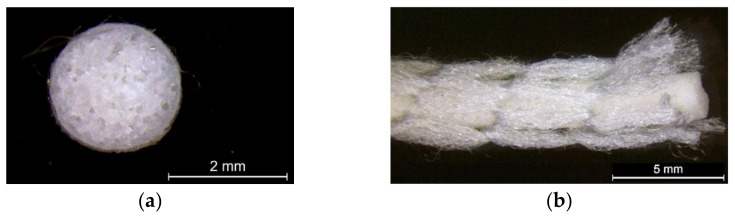
Microscopic view of (**a**) cross section of silicone foam rod, and (**b**) side view of foam rod wrapped by the net.

Silicone foam rods with a diameter of 2.25 mm were used as the inlay as they are flexible enough to be laid into the fabric during the knitting process. Prior to knitting, the inlaid material was wrapped by using a knitted net to reduce the surface friction during insertion. The net was knitted by using a multifunction twisting machine for fancy yarn (SFM32-04, Kunshan Shun Feng Textile Co., LTD., Kunshan, China) with 4 needles. The yarn used was 2 ends of DRYARN^®^ 140D 100% polypropylene yarn (Aquafil S.P.A., Via Linfano, Arco, Italy). The foam rods were then manually inserted into the knitted net. A microscopic view of the knitted nets and inserted silicone foam rods is provided in Figure 1.

### 2.2. Inlaid Spacer Fabric Samples

**Figure 2:**
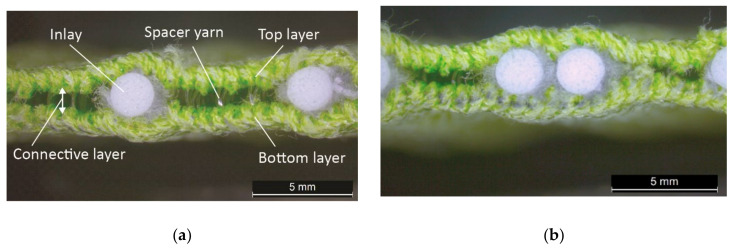
Microscopic view of spacer fabric with (**a**) random inlays and (**b**) inlays with localized orientation.

Ten spacer fabric samples with different inlay densities, inlay orientation and spacer yarn density were fabricated, and two knitted fabrics without inlays were used as the control. All of the fabric samples were knitted on a 14-gauge V-bed flat knitting machine (SVR123SP, SHIMA SEIKI, Wakayama, Japan) equipped with yarn feeder tips (4.5D) which are suitable for inserting materials with a diameter below 4 mm. Two ends of the 1/27NM 63% cupro 37% polyester and one end of 107D high power lycra were used as the yarn for the surface layer in a single jersey structure. The connective layer was constructed by using one end of 100% 220D polyester monofilament. All the samples were prepared by using the same knitting tension and parameters. Two of the same samples were made for each knitting condition. The design of experiment and details of the sample specifications are provided in Table 1 and Table 2. A random inlay orientation means that the inlaid foam rods are randomly placed in all directions, while localized orientation means a continuous inlay as shown in Figure 2. The knitting notations of the fabric are provided in Table 3.

### 2.3. Evaluation of Mechanical and Thermal Behaviours of Fabric

With reference to the key requirements and end-uses of insoles including cushioning purposes, resistance to body weight and wear comfort during walking, tests on the physical, compression and thermal comfort properties of the samples were conducted (Table 4). The fabric samples were conditioned for 24 h at a temperature of 20 ± 1 °C and relative humidity of 65 ± 5% before the testing took place. Depending on the sample size, the tests were repeated 4 to 6 times on different areas of the samples, and their mean value was calculated and used.

#### 2.3.1. Physical Properties

The fabric thickness was measured under a pressure of 4 gf/cm^2^ with an accuracy of 0.01 mm. A 3D-optical microscope was used to examine the variations in the surface unevenness of the samples. The air permeability test was carried out under a water pressure difference of 125 Pa.

#### 2.3.2. Compression and Impact Force Reduction

**Figure 3:**
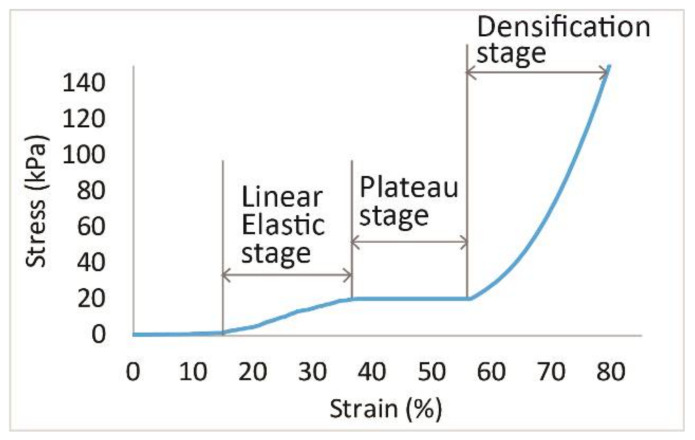
Plotted compression stress-strain of spacer fabric.

Each fabric sample was tested by using a compression tester with a flat circular indenter that is 150 mm in diameter. The compression test was conducted at a rate of 12 mm/min and the samples were compressed up to 80% of their initial thickness. As shown in Figure 3, the compression process of the spacer fabrics can be divided into three stages including the linear elasticity (Stage I), plateau (Stage II), and densification (Stage III) stages [12,25]. In Stage I, the presence of a smaller slope is observed as the compressed monofilaments start to buckle and shear constantly at a larger scale. When the fabrics are further compressed in Stage II, a nearly constant stress can be observed. This shows the collapse of the spacer fabric structure and inlaid material as the compression strain of the fabric is largely increased under a small increase in stress. In Stage III, the structure completely collapses in which the monofilaments and the inlays come into contact with each other and the outer layers. The sample becomes stiff and rigid so the slope of the curve becomes steep again. A higher maximum compression stress means that the fabric can withstand a higher compression force. The strain is calculated as the distance of the compression by the indenter divided by the original thickness of the specimen. A steeper curve in Stage I represents stiffer mechanical behaviour of the fabric with a higher Young’s modulus.

When measuring the impact force reduction, two specimens of each sample were stacked on top of each other with dimensions of 100 mm × 100 mm before the testing was carried out. A ball bearing was released and dropped onto the sample through a straight tube at a height of 400 mm. The highest impact force was measured by using a load cell placed at the bottom of the instrument. The impact force reduction capacity of the sample is defined as a percentage of the maximum impact force with the sample and the ground surface [26]:

(1) $${FR}_{x}=\left(1-\left(\frac{F_{x}}{F_{o}}\right)\right)\times 100\%,$$

where *FR_x_* is the impact force reduction of the sample (%), *F_x_* is the peak force measured for the sample (N), and *F_o_* is the peak force measured for the ground surface.

#### 2.3.3. Thermal and Evaporative Resistance

The thermal and evaporative resistance tests were conducted in an air temperature of 25 °C at a relative humidity of 65% with air flowing over the hot plate and air velocity of 1 m/s. The test began when the backside of the fabric was placed onto the hot plate to simulate contact with human skin. All wrinkles were eliminated from the fabric to prevent air bubbles from forming between the fabric sample and the hot plate. The average intrinsic thermal resistance of the fabric is calculated by using:

*R_cf_* = ((*T_plate_* − *T_air_*) *A*/*H_c_*) − *R_b_*, (2)

where *R_cf_* is the intrinsic resistance to dry heat transfer provided by the fabric system (m^2^·K/W), *T_plate_* is the surface temperature of the hot plate (°C), *T_air_* is the air temperature (°C), *A* is the area of the tested section on the hot plate (m^2^), *H_c_* is the power input (W), and *R_b_* is the thermal resistance of the still air where no fabric was placed onto the plate (m^2^·K/W).

For the evaporative resistance test, the sweat pores of the hot plate emitted water to the surface of the plate and a liquid barrier was used to cover the plate to prevent wetting of the fabric specimens. The liquid barrier was adhered closely to the hot plate and guard section with no wrinkles or air bubbles present. Then, a fabric sample was placed on the hot plate. The average intrinsic evaporative resistance of the fabric sample is calculated as follows:

*R_ef_* = ((*P_plate_* − *P_air_*) *A*/*H_e_*) − *R_e_*, (3)

where *R_ef_* is the intrinsic resistance to evaporative heat transfer provided by the fabric system (m^2^·K/W), *P_plate_* is the water vapor pressure at the plate surface (kPa), *P_air_* is the water vapor pressure in the air (kPa), *A* is the area of the section in which the testing was carried out on the hot plate (m^2^), *H_e_* is the power input (W), and *R_e_* is the evaporative resistance value measured for the air layer and liquid barrier with no fabric placed onto the hot plate (m^2^·K/W).

#### 2.3.4. Statistical Analysis

The experimental data were analysed by using SPSS 23 (IBM Corp., Armonk, NY, USA). A multivariate analysis of variance (MANOVA) was used to examine the mean differences among the three different independent variables: (1) inlay density, (2) orientation of inlays and (3) spacer yarn density on five dependent variables (maximum compressive stress, impact force reduction, air permeability, and thermal and evaporative resistance). Prior to the analysis, the values were evaluated to ensure that the assumptions for the multivariate tests are valid. Measures of skewness and kurtosis, histograms and normal Q-Q plots were used for the dependent and independent variables. Observation of these measures and plots showed a normal distribution at the different levels of these variables. The significance level of the statistical analysis was set at 0.05.

## 3. Results and Discussion

### 3.1. Air Permeability

**Figure 4:**
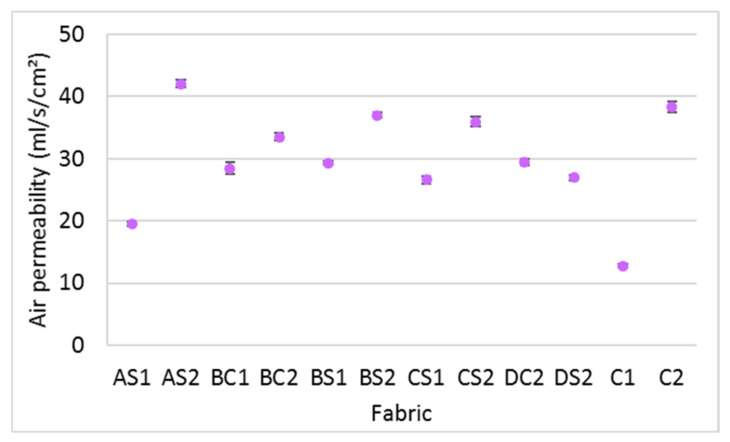
Air permeability of the inlaid spacer fabric and controls.

This study found that over 99 percent of the variance in air permeability is accounted for by the spacer yarn density (η^2^ = 0.996) and inlay density (η^2^ = 0.991) but no significant difference is found with the orientation of the inlays (*p* > 0.05). It is interesting that the fabrics with a higher spacer yarn density (AS2, BC2, BS2, CS2 and C2) have a relatively higher air permeability than those with less spacer yarn (AS1, BC1, BS1, CS1 and C1). Additional pulling forces are applied to the surface layer when the spacer yarn density of fabric is increased. The knitted loops of the surface layer are then extended so that the restriction of air flow through the fabric is reduced. This is confirmed by the lower stitch density and fabric weight of the spacer fabrics with a higher spacer yarn density (AS2, BS2, CS2 and C2) (Table 5). When comparing the various inlay densities (AS2, BS2, CS2 and DS2), the samples with higher inlay density exhibit relatively lower air permeability (Figure 4). The reason is that the inlaid foam rods block the circulation of trapped air between the two surface layers.

### 3.2. Compression Behaviour

**Figure 5:**
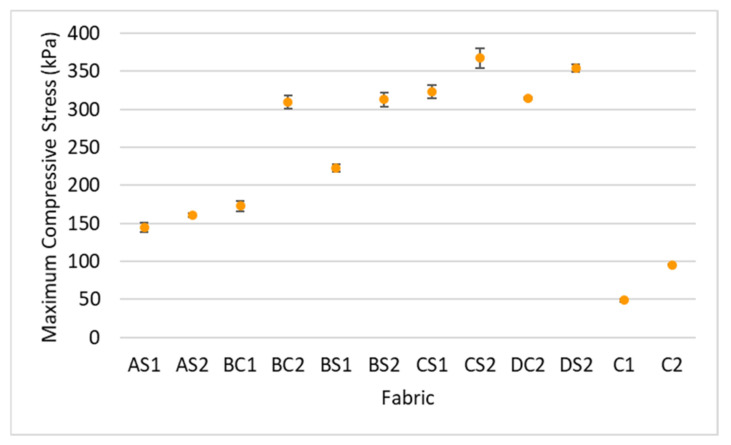
Maximum compressive stress of inlaid spacer fabrics and controls.

The results of the factorial MANOVA show an overall significant difference between the maximum compressive stress, various inlay densities (F = 486.97, *p* < 0.001, and η^2^ = 0.993), spacer yarn density (F = 335.18, *p* < 0.001, and η^2^ = 0.971) and orientation of the inlays (F = 65.14, *p* < 0.001, and η^2^ = 0.867). The inlay and spacer yarn densities account for over 97 percent of the variance in the maximum compressive stress while the orientation of the inlays has the least impact and accounts for only 87% of the variance. As shown in Figure 5, all of the spacer fabric samples with inlay have a higher maximum compressive stress versus the fabric samples without inlay (C1 and C2). This is agreement with the findings in Yu et al. [12], in that inlays largely strengthen spacer fabric to withstand the compressive stress.

#### 3.2.1. Effect of Inlay Density

**Figure 6:**
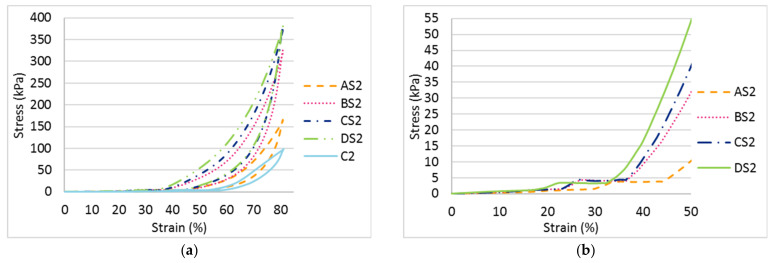
Compression stress-strain curves of the inlaid spacer fabrics with different inlay densities: (**a**) at 80% strain and (**b**) at 50% strain.

**Figure 7:**
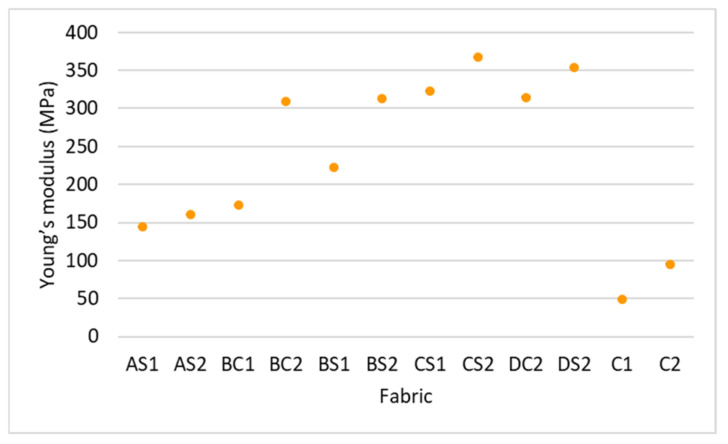
Young’s modulus of the inlaid spacer fabric and controls.

A higher maximum compressive stress can be observed when the inlay density increases from 1.2 course/cm (AS2) to 2.5 course/cm (DS2); see Figure 6a. Meanwhile, the Young’s modulus of the fabric generally increases with inlay density (Figure 7). This means that the spacer fabric with more inlays has stiffer mechanical behaviour and higher compression resistance. The stress-strain curve in Figure 6 shows that the area under the curve is the energy absorbed by the fabric. In Stage III, the area under the curve of DS2 is more than twice that of C2, the fabric sample without inlay (Figure 6a). This shows that the inlaid foam rods absorb a greater amount of energy when they deform and increase the strength of the spacer fabric to resist compression stress.

It is interesting that Stage II appears at a similar compression stress of around 3.81 kPa and a similar range of compression strain (around 10.45%) is obtained when the inlay density is increased; see Figure 6b. However, Stage II appears at a lower level of strain for fabric with more inlays (1.36 mm in DS2, and 1.86 mm in AS2). In Stage II, the wider range of compression strain and higher stress mean that the material has a better cushioning effect by absorbing more energy before reaching Stage III. Stage II which appears at a lower level of strain means less distortion of the fabric occurs under the same compression stress. Although the fabric samples with various inlay densities absorb a similar amount of energy in Stage II under a low stress level, fabric with the highest inlay density (DS2) has less deformation under the same stress. This may be due to the surface unevenness of the fabric.

**Figure 8:**
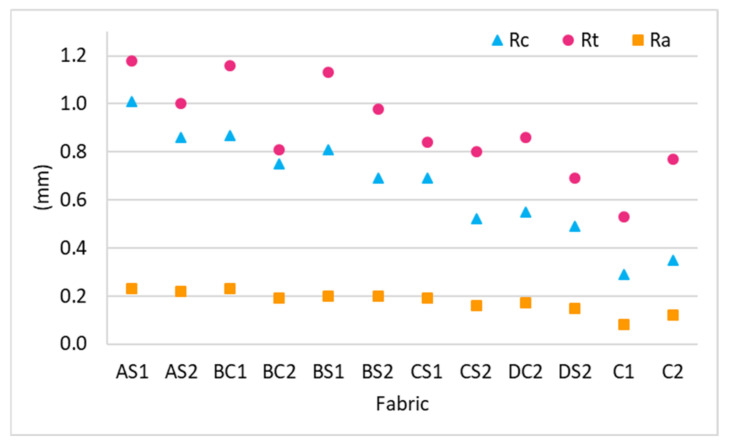
Surface unevenness Rc, Rt and Ra of the inlaid spacer fabrics and controls.

**Figure 9:**
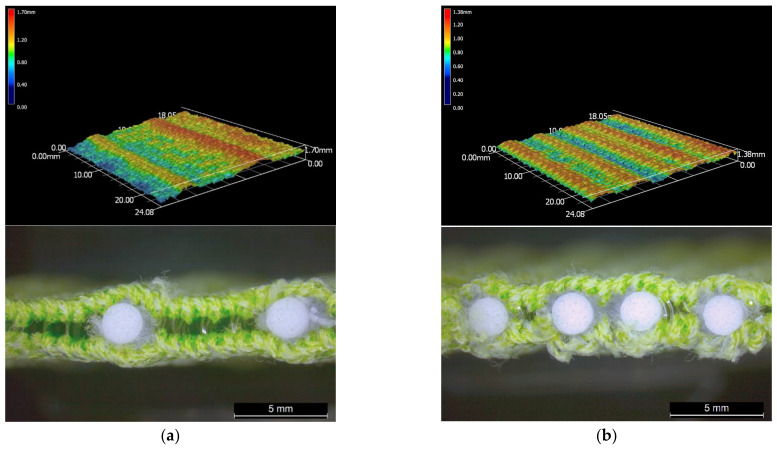
Microscopic view and surface thickness variations of inlaid spacer fabrics (**a**) AS2 and (**b**) DS2 along the wale direction.

**Figure 10:**
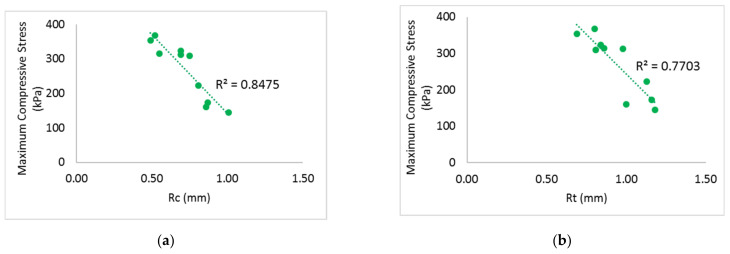

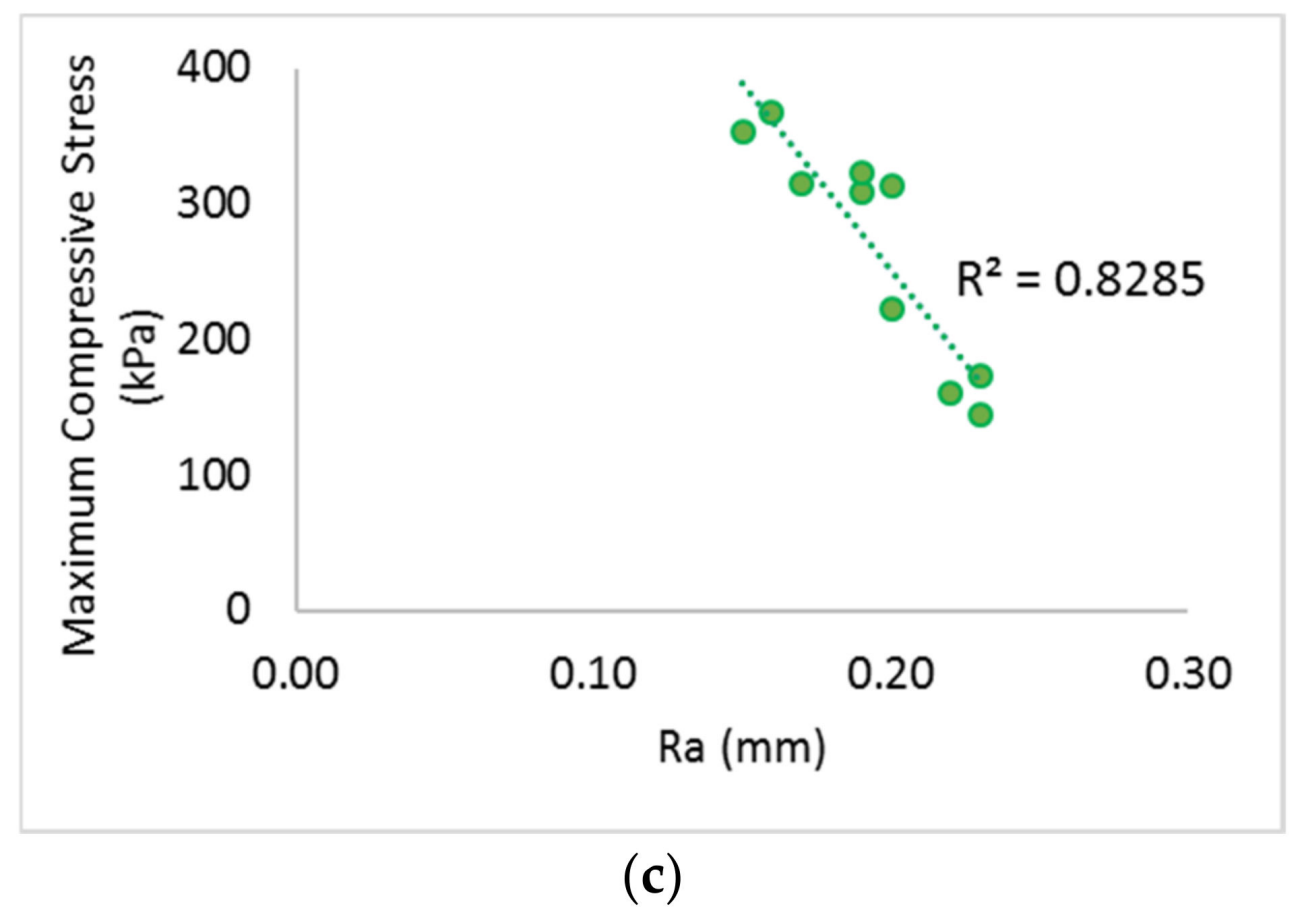
Linear relationship between maximum compressive stress and surface unevenness: (**a**) Rc, (**b**) Rt and (**c**) Ra.

Rc, Rt and Ra have a small value which indicates that the surface layer is uneven. As shown in Figure 8, the fabric unevenness can be reduced by increasing the inlay density. The surface of DS2 is relatively flat with a lower Rc, Rt and Ra while AS2 is comparatively more wavy (Figure 9). The compressive forces can be evenly distributed throughout the entire surface of DS2 which increases the compression resistance but this is only true for the convex areas of AS2. Therefore, the linear relationship between surface unevenness and maximum compressive stress can be found in Figure 10. The reduced surface unevenness (Rc, Rt and Ra) of the fabric sample results in an increased maximum compressive stress. Therefore, the spacer fabric with more inlays can withstand higher compressive forces due to the even distribution of the force on the flat surface and the increased fabric stiffness. The materials used for insoles should be less susceptible to deformation under the same stress to enhance standing and walking stability to the same degree as a hard insole.

#### 3.2.2. Effect of Spacer Yarn Density

**Figure 11:**
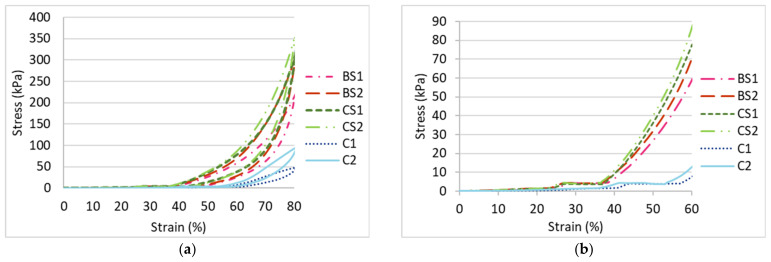
Compression stress-strain curves of inlaid spacer fabrics with different spacer yarn densities: (**a**) at 80% strain and (**b**) at 60% strain.

As shown in Figure 7 and Figure 11, the spacer fabric samples with a higher spacer yarn density (BS2, CS2 and C2) have a higher maximum compression stress and higher Young’s modulus than the samples with a lower spacer yarn density (BS1, CS1 and C1) respectively. This shows that the additional spacer yarns in the connective layer provide extra support to the spacer fabric structure, as more stress is needed to compress the fabric and the extra spacer yarns also increase the stiffness of the fabric. However, the curves of BS2 and CS1 are similar (Figure 11a), which means that the fabric sample with 2 inlay courses and 10 spacer yarn courses per cm (BS2) has similar compression properties as the fabric sample with 2.3 inlay courses and 4 spacer yarn courses per cm (CS1).

Similar to the effect of the inlay density, Stage II of the fabric samples with various spacer yarn and inlay densities (BS1, BS2, CS1, CS2, C1 and C2) appears at a similar compression stress (around 4 kPa) with similar range of strain (around 11.11%) (Figure 11b). However, Stage II of the inlaid spacer fabric appears at a compression strain of 27% which is smaller than the control fabrics (42.26%). Although the inlaid spacer fabric absorbs a similar amount of energy under a low stress condition, the deformation of the spacer fabric structure with inlays is 15.26% less than that of the fabric without inlays under a compression stress of 4 kPa.

#### 3.2.3. Effect of Inlay Orientation

**Figure 12:**
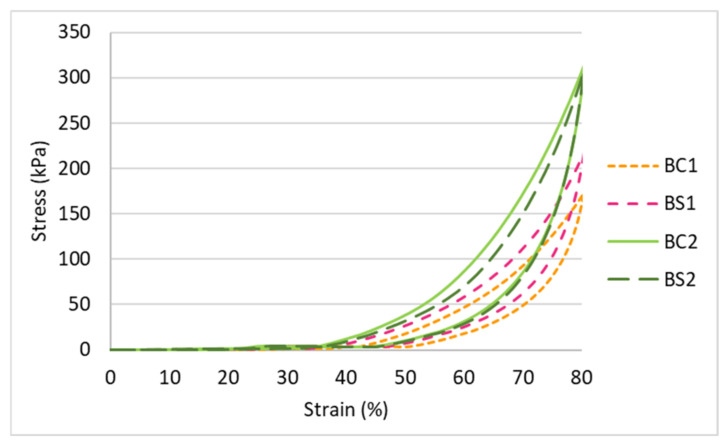
Compression stress-strain curves of inlaid spacer fabrics with different orientation of the inlays.

As shown in Figure 7 and Figure 12, the curve of BS1 indicates a higher maximum compressive stress and Young’s modulus as opposed to BC1. However, BC2 and BS2 have similar compression properties as they have a similar curve. This means that the compression properties are correlated with both inlay orientation and spacer yarn density which validates the results of the MANOVA. The fabric sample with inlays in a random arrangement with a lower spacer yarn density (BS1) is more rigid and has a higher compression resistance in comparison to the inlay in a localized arrangement (BC1). As for the fabric samples with a higher spacer yarn density (BC2 and BS2), the effect of the inlay orientation on the compression properties becomes less significant.

### 3.3. Impact Force Reduction

**Figure 13:**
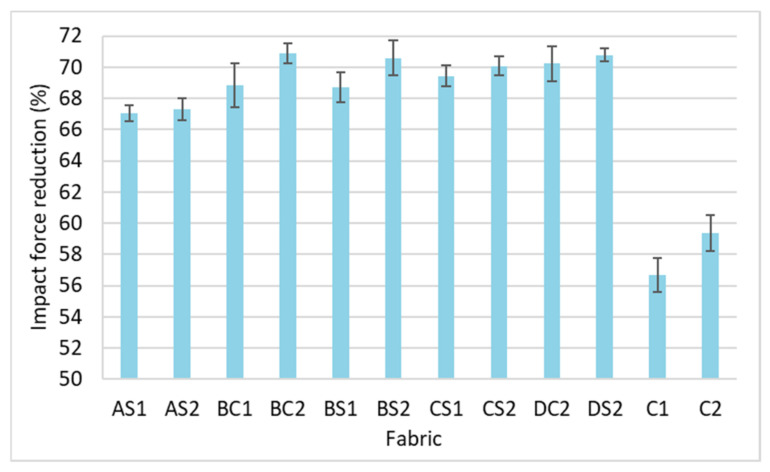
Percentage of impact force reduction of inlaid spacer fabrics and controls.

Fabric that can sustain a higher impact force is a more suitable material for insoles which would reduce the plantar pressure of the foot. As shown in Figure 13, all of the inlaid spacer fabrics have a higher force reduction than the spacer fabrics without inlays (C1 and C2). This shows that the inlaid rods enhance the ability of the fabric to reduce the impact forces. Among the inlaid spacer fabric samples, the sample with the higher inlay density can generally reduce higher impact forces. The fabric samples with a higher spacer yarn density (AS2, BS2, CS2 and C2) show a higher reduction of impact forces than those with less spacer yarn (AS1, BS1, CS1 and C1). The ability to reduce higher impact forces is a function of the higher inlay and spacer yarn densities. These results are confirmed by a MANOVA analysis, and significant differences are observed only between the inlay density (F = 10.89, *p* < 0.005, and η^2^ = 0.766) and spacer yarn density (F = 5.32, *p* < 0.05, and η^2^ = 0.347) on the impact force reduction. Around 77% of the variance in force reduction is accounted by the inlay density while the spacer yarn density only accounts for 35% of the variance. This implies that the inlay density is the main factor in reducing the impact forces.

### 3.4. Thermal Comfort Properties

Unlike the knitted flat fabrics [27], the results of the Pearson’s correlations indicated that the surface unevenness—Rc, Rt, and Ra, has no significant correlation with thermal and evaporative resistance (*p* > 0.05).

#### 3.4.1. Evaporative Resistance

**Figure 14:**
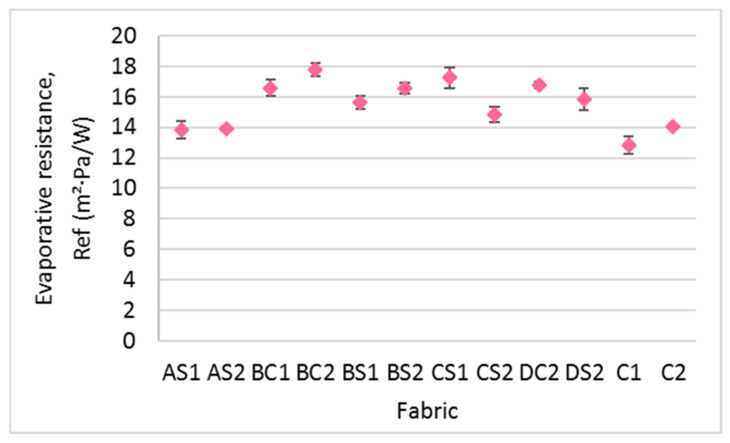
Evaporative resistance of the inlaid spacer fabrics and controls.

Unlike air permeability, the evaporative resistance of the fabric samples depends on the inlay density (F = 32.32, *p* < 0.001, and η^2^ = 0.907) and orientation of the inlays (F = 14.98, *p* < 0.005, and η^2^ = 0.600) but not the spacer yarn density (*p* > 0.05). When comparing AS1, BS1 and CS1, the evaporative resistance of the inlaid spacer fabric samples increases with inlay density (Figure 14). The fabric samples with inlays in a random arrangement (BS1, BS2, and DS2) have lower evaporative resistance than those with a continuous inlay arrangement of the foam rods (BC1, BC2, and DC2). This is likely because the inlaid material arranged in a localized area provides extra resistance to inhibit the moisture from infiltrating the top surface layer to the bottom surface layer. In insole applications, it is recommended that the foam rods are inlaid in a random arrangement to allow optimal moisture evaporation and reduce the likelihood of ulceration and discomfort from humidity inside the shoes.

#### 3.4.2. Thermal Resistance

**Figure 15:**
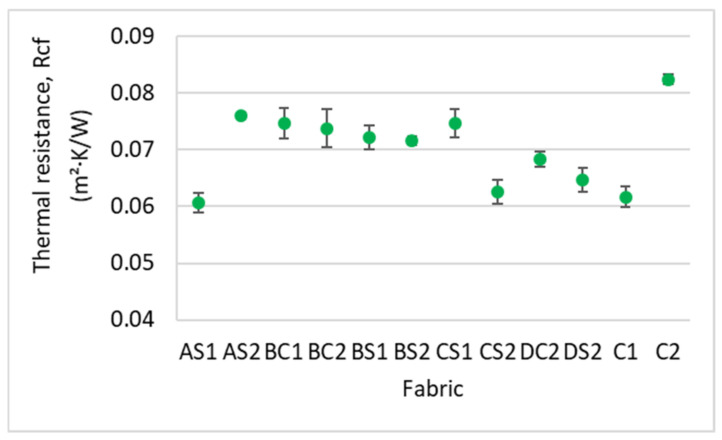
Thermal resistance of the inlaid spacer fabric samples and controls.

The thermal properties of insole material should accommodate the environment of the end use of the insole. In cold environments, insole material with higher thermal resistance should be used to keep the wearer warm. As with air permeability, the thermal resistance of the spacer fabric samples is significantly affected by the inlay density (F = 6.27, *p* < 0.05, and η^2^ = 0.653) and spacer yarn density (F = 14.286, *p* < 0.005, and η^2^ = 0.588) only; however, they account for less than 66% of the variance. When comparing the fabric samples with a low spacer yarn density (AS1, BS1 and CS1) (Figure 15), the fabric sample with a higher inlay density exhibits a higher thermal resistance as the inlaid foam rods prevent air from passing through the fabric. However, for the fabric samples with a high spacer yarn density, the thermal resistance generally decreases with increased inlay density (AS2, BS2 and CS2). This is due to the reduced stitch density of the surface layers resultant of additional spacer courses. In agreement with the findings of Muthu Kumar et al. (2020), ease of passage of air is facilitated with lower stitch density which leads to lower thermal resistance [8]. Along the same line of reasoning, the fabric samples with a higher spacer yarn density (BC2, BS2 and CS2) have lower thermal resistance in comparison to BC1, BS1 and CS1.

## 4. Conclusions

Traditional insoles are primarily designed and made of a plurality of materials with various stiffnesses, compression resistances and thermal comfort properties that correspond to the 3D shape and localised pressure at different regions of the plantar of the foot. The process of constructing such insoles is highly complex and time-consuming. A novel knitted spacer fabric structure with inlays is proposed in this study which can greatly simplify the insole fabrication process with a one-off knitting approach. A thorough understanding of the structural parameters that affect the functional performance and suitability of the fabric during practical use could have clinical significance for advancing the design process of insoles with better foot protection and wear comfort. In this study, CS2 is the optimal inlaid spacer fabric for insoles application. It can withstand high compressive stress, reduce high impact forces, air permeable and allow optimal heat and moisture evaporation. The effect of the inlay density, orientation of the inlays and spacer yarn density on the mechanical and thermal behaviours of the fabric has been systematically investigated. The following conclusions are drawn based on the experimental results.

- The high spacer yarn density of inlaid spacer fabric offers good air permeability. The size of the knitted loops on the surface layer are extended by the additional spacer yarn that allow air to flow through the material. Therefore, spacer fabric with a higher spacer yarn density has higher air permeability, and lower stitch density and fabric weight;
- For the mechanical properties, the compression resistance of the inlaid spacer fabric is largely increased by an increased number of inlays and spacer yarns while the absorbed compression energy in Stage II remains unchanged. The increased compression resistance is associated with the even distribution of forces exerted onto the flat surface of the fabric and the increased fabric stiffness. The effect of the inlay orientation on the compressive stress is less significant in this study. The fabric samples made with inlaid yarns embedded in a random orientation are stiffer than those with inlaid yarns embedded in a localized orientation;
- Meanwhile, spacer fabrics with higher inlay density and more spacer yarns can reduce higher impact forces. The inlay density is the key factor as the inlay foam rods can effectively reduce and absorb the impact forces for cushioning;
- The evaporative resistance performance of the inlay spacer fabrics is increased with inlay density and embedded in a localized orientation. On the other hand, the fabric thermal resistance is decreased with the higher inlay density for fabric with a high spacer yarn density. Samples with more spacer yarn have a lower stitch density in the surface layers which facilitates air passage, thus leading to low thermal resistance. Therefore, samples with more spacer yarns while embedded in a random orientation allow optimal moisture evaporation and reduce thermal discomfort of humidity to the wearers.

## Figures and Tables

**Table 1:** Design of experiment.

Factor	Level			
Inlay density (ratio of spacer and inlay structure)	4:1	3:2	1:1	2:3
Inlay Orientation	Random	Localized		
Number of spacer yarn per spacer structure	1 course	2 courses		

**Table 2:** Sample specifications of inlaid spacer fabrics.

pslp	Knitted Structure	Inlay Density (Course per cm)	Orientation of Inlay	Spacer Yarn Density (Course per cm)
AS1	A	1.20	Random	5
AS2	B	1.20	Random	10
BC1	C	2.00	Localized	5
BC2	D	2.00	Localized	10
BS1	E	2.00	Random	5
BS2	F	2.00	Random	10
CS1	G	2.30	Random	4
CS2	H	2.30	Random	8
DC2	I	2.50	Localized	10
DS2	J	2.50	Random	10
C1	C1	Nil	Nil	13
C2	C2	Nil	Nil	26

**Table 3:** Knitting notations and images of fabrics with different knitted structures.

Fabric	AS1	AS2	BC1	BC2	BS1	BS2	CS1	CS2	DC2	DS2
Structure	A	B	C	D	E	F	G	H	I	J
Knitting notation	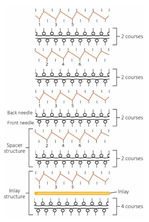	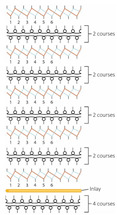	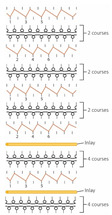	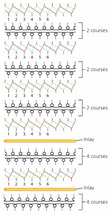	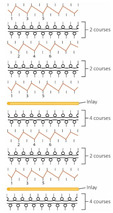	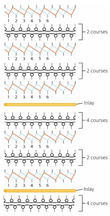	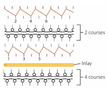	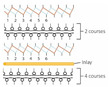	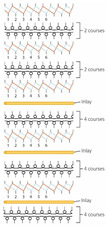	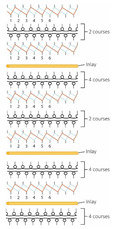
Image	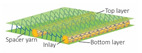	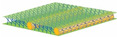	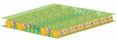	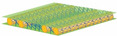	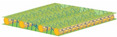	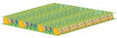	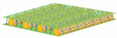	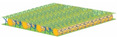	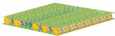	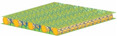

**Table 4:** Summary of test methods.

Property	Device	Testing Standard
Thickness	Dial thickness gauge (Model H, Peacock OZAKI MFG. Co., Ltd., Tokyo, Japan)	ASTM D1777 Standard Test Method for Thickness of Textile Materials
Hardness	Durometer (GS-744G, Type: FO, TECLOCK Co., Ltd., Nagano, Japan)	ASTM D2240-05: 2010 Standard Test Method for Rubber Property—Durometer Hardness
Surface unevenness	3D-optical microscope (VR-3000, KEYENCE, Osaka, Japan)	ISO4287:1997 Surface unevenness-Definitions
Air permeability	Air permeability tester (SDL M021S, SDL International Textile Testing Solutions, Rock Hill, SC, USA)	ASTM-D737 Standard Test Method for Air Permeability of Textile Fabrics
Compression	Compression tester (Instron 4411, Instron, Norwood, MA, USA)	ASTM D575 Standard Test Methods for Rubber Properties in Compression
Thermal and evaporative resistance	Sweating guarded hot plate (YG(B)606G, Wenzhou, China)	ASTM F1868-17 Standard Test Method for thermal and evaporative resistance of clothing materials

**Table 5:** Physical properties of the inlaid spacer fabrics.

Sample Code	Weight (g/m^2^)		Thickness (mm)		Stitch Density (loop/cm^2^)		Hardness (Shore A)	
	Mean	SD	Mean	SD	Mean	SD	Mean	SD
AS1	1483.32	6.12	4.30	0.03	93.33	4.04	83.17	1.33
AS2	1417.25	0.46	4.31	0.03	91.00	0.00	86.17	1.72
BC1	1649.50	18.62	4.34	0.03	93.33	4.04	86.17	0.93
BC2	1683.28	15.94	4.39	0.05	91.00	0.00	86.50	1.22
BS1	1669.15	6.38	4.40	0.04	91.00	0.00	87.33	0.82
BS2	1620.13	21.14	4.39	0.05	86.50	3.91	87.67	0.82
CS1	1759.97	17.31	4.35	0.04	88.83	3.75	88.67	1.21
CS2	1689.88	17.47	4.39	0.03	86.33	4.04	88.83	0.41
DC2	1866.20	7.60	4.34	0.05	82.33	3.75	88.92	0.92
DS2	1861.70	22.59	4.33	0.04	82.33	3.75	89.00	0.84
C1	1094.38	5.65	3.51	0.05	108.00	4.00	88.67	1.37
C2	1068.20	16.54	3.75	0.14	106.67	4.62	89.00	0.63
